# COVID-19 and strongyloidiasis: what to expect from this coinfection?

**DOI:** 10.6061/clinics/2021/e3528

**Published:** 2021-11-10

**Authors:** Carolina Victoria Marcitelli Pereira, Giovanna Ribeiro Achur Mastandrea, Ana Clara Cassine de Souza Medeiros, Ronaldo Cesar Borges Gryschek, Fabiana Martins de Paula, Marcelo Andreetta Corral

**Affiliations:** I Faculdade de Medicina, Universidade Santo Amaro, Sao Paulo, SP, BR.; II Laboratorio de Investigacao Medica (LIM06-Laboratorio de Imunopatologia da Equistossomose), Hospital das Clinicas HCFMUSP, Faculdade de Medicina, Universidade de Sao Paulo, Sao Paulo, SP, BR.; III Secao de Helmintologia, Instituto de Medicina Tropical (IMT), Faculdade de Medicina FMUSP, Universidade de Sao Paulo, Sao Paulo, SP, BR.

Strongyloidiasis is a parasitic infection caused by the intestinal nematode *Strongyloides stercoralis*. It is a chronic and asymptomatic parasite in most immunocompetent individuals. In immunocompromised patients, especially those undergoing corticotherapy, the disease can progress to advanced stages, such as hyperinfection syndrome and disseminated disease (1). Corticosteroids are metabolized in the liver to chemical compounds structurally similar to ecdysone and are used by helminths for oviposition and larval ecdysis stimulation, promoting an accelerated autoinfection cycle (2).

The coronavirus disease (COVID-19) pandemic remains a health challenge for society worldwide. The advancing knowledge of the physiopathology suggests that a rapid replication of severe acute respiratory syndrome coronavirus 2 (SARS-CoV-2) may lead to an exacerbated immune response involving many cytokines (cytokine storm), triggering a severe inflammatory response in the lungs. Using corticosteroids and anticoagulants in such cases has produced beneficial results (3-5), with dexamethasone and methylprednisolone being used most frequently (6-7).

The impact of the COVID-19 pandemic on *S. stercoralis* infection remains to be elucidated. However, there are few reports of severe strongyloidiasis in patients with COVID-19 after undergoing treatment with corticosteroids (8-12); currently, only six cases of coinfection with *S. stercoralis* and COVID-19 have been reported (Figure 1). Clinical data of these patients are incipient or partial; however, generally, the majority of the reported cases tended to be in male individuals (66.7%), with an average age of 61.3 years. These individuals were admitted to the hospital for COVID-19 treatment, were released from the hospital, and returned after 22.8 days on average with skin signs and symptoms, such as dermatitis, followed by respiratory and intestinal manifestations. The diagnosis of *S. stercoralis* infection was confirmed by parasitic stool examination in one case (16.7%), enzyme-linked immunosorbent assay in two (33.3%), microscopic stool and serological methods in two (33.3%), and microscopic sputum in one (16.7%).

Different therapeutic regimens (Figure 1) were used to treat SARS-CoV-2 infection with methylprednisolone and dexamethasone. In parallel with corticosteroids, antiviral drugs were also used. For *S. stercoralis* infection, ivermectin was used in 50% of the patients, albendazole in 16.7%, and the combination of both in 33.3%. All patients survived COVID-19 and strongyloidiasis. It is important to highlight that 83.3% of the described cases occurred in non-endemic areas (1,13) for *S. stercoralis* infection, possibly indicating a link to immigration flows.

Oliveira (6) reported the importance of the identification of *S. stercoralis* infection using diagnostic methods for parasite detection, especially cultures, or even serological screening. Once the helminth parasite is detected, anti-helminthic treatment is recommended before corticosteroid therapy is administered. De Wilton et al. (5) proposed an algorithm for screening the risk of *S. stercoralis* exposure in patients samples with COVID-19 at a hospital in England. In general, all patients who had travelled or immigrated from endemic areas and those with a high risk of *S. stercoralis* infection underwent a diagnostic test and treatment with ivermectin. Although England is not considered an endemic area for *S. stercoralis* infection, there is a concern about the development of severe forms of this parasitosis when associated with corticosteroid therapy, especially in immigrants.

Unlike in the countries that reported cases of coinfection, the reality of strongyloidiasis in Brazil is distinct, with an estimated occurrence varying between 5.5% and 20% based on the microscopic parasitic examination of stool. When other diagnostic methods are analyzed, these numbers may be significantly higher since directly detecting larval forms is not needed (13,14). Pandemic situations, such as COVID-19, bring awareness to the scientific community worldwide to the need for studies on other non-viral infectious agents for the prevention of fatal consequences of possible associations. In this scenario, the possibility of asymptomatic infection by *S. stercoralis* becoming symptomatic and severe with the use of corticosteroids is evident.

There is a need to apply and to implement more sensitive and specific diagnostic techniques for strongyloidiasis that could be used in immunocompetent and immunocompromised individuals (5,6). The public health systems need to consider the construction and validation of a diagnostic protocol. Many diagnostic methods have been reported in the literature (15) based on the direct detection of the parasite by agar plate culture or molecular methods, or even the indirect detection of antibodies, antigens, or immune complexes; however, none of these methods are available in the Brazilian national health system, only for research. Therefore, strongyloidiasis continues to be considered one of the most neglected parasitic diseases worldwide. The rapid identification of positive cases could minimize the occurrence of severe cases resulting from coinfection; in Brazil, this would demonstrate the real situation of the condition in the country since it has high incidence rates of this important parasitosis.

## Figures and Tables

**Figure 1:**
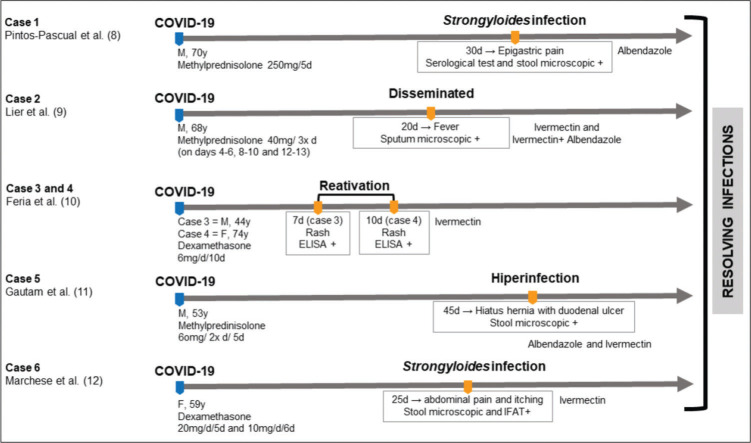
Timeline of the main events related to SARS-CoV-2 and *S. stercoralis* coinfections.
